# Dapsone-Induced Systemic Drug Reaction in a Patient With Immunoglobulin A Vasculitis

**DOI:** 10.7759/cureus.106449

**Published:** 2026-04-05

**Authors:** Luke Hamilton, Antonio R Jimenez, Sarah Kesaria, Anthony Linfante

**Affiliations:** 1 John Sealy School of Medicine, University of Texas Medical Branch, Galveston, USA; 2 Department of Dermatology, University of Texas Medical Branch, Galveston, USA; 3 Department of Dermatopathology, University of Texas Medical Branch, Galveston, USA

**Keywords:** dapsone hypersensitivity syndrome, drug hypersensitivity reaction, drug reaction with eosinophilia and systemic symptoms (dress) syndrome, immunoglobulin a vasculitis, systemic dapsone

## Abstract

Dapsone is widely used for inflammatory dermatoses and vasculitides but may cause severe systemic hypersensitivity reactions. Diagnosis can be challenging when clinical features overlap with underlying inflammatory disease and when eosinophilia is absent. We report a case of a young man with biopsy-confirmed immunoglobulin A vasculitis (Henoch-Schönlein purpura) who developed facial and neck edema, a diffuse morbilliform eruption, and hepatocellular liver injury approximately four weeks after initiation and dose escalation of dapsone. Laboratory evaluation demonstrated elevated aminotransferases with normal creatinine and absent eosinophilia, along with anemia and reticulocytosis suggestive of concurrent dapsone-related hematologic toxicity. The patient was receiving ongoing cyclosporine and had recent high-dose corticosteroid exposure at the time of presentation. Dapsone was discontinued and systemic corticosteroids were administered with rapid clinical improvement. This case highlights diagnostic pitfalls in recognizing severe drug reactions in immunosuppressed patients and emphasizes the importance of integrating medication timelines, organ involvement, and immunosuppression status when evaluating suspected drug reactions.

## Introduction

Dapsone is an anti-inflammatory sulfone commonly prescribed for neutrophilic dermatoses, autoimmune blistering disorders, and vasculitides. Although generally well tolerated and efficacious in many patients, clinicians must remain mindful of its potential for serious adverse reactions including hemolysis, methemoglobinemia, agranulocytosis, and systemic hypersensitivity reactions [1]. Severe reactions historically termed dapsone hypersensitivity syndrome are now recognized within the broader spectrum of drug reaction with eosinophilia and systemic symptoms (DRESS), a delayed drug reaction characterized by rash, facial edema, hematologic abnormalities, and internal organ involvement, most commonly hepatitis [2-4]. These reactions are uncommon but clinically significant due to their potential severity and associated morbidity.

Recognition of systemic drug reactions can be particularly difficult when cutaneous findings overlap with an underlying inflammatory disorder or when hallmark laboratory features such as eosinophilia are absent. Contemporary literature emphasizes that these reactions represent a clinical spectrum rather than a rigid laboratory-defined entity and that early or immunosuppression-modified presentations may not fulfill classic criteria [3-6]. Cases of DRESS without eosinophilia have been described, particularly early in the disease course or in patients receiving immunosuppressive therapy. We present a case of dapsone-induced systemic drug reaction in a patient with biopsy-proven immunoglobulin A vasculitis (Henoch-Schönlein purpura), highlighting diagnostic pitfalls, differential diagnosis, and practical management considerations.

## Case presentation

**Figure 1:**
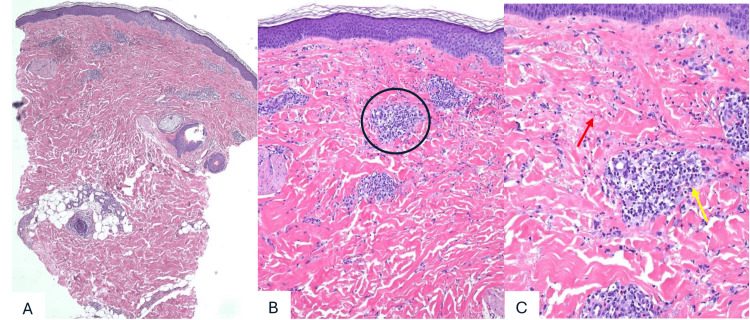
Histopathologic findings of immunoglobulin A vasculitis from a skin biopsy of the right lower leg. (A) Low-power view showing superficial and deep perivascular inflammation in the dermis (hematoxylin and eosin stain, x4). The black circle highlights an area of perivascular inflammation. (B) Medium-power view demonstrating perivascular neutrophilic infiltrates with leukocytoclasia and associated extravasated erythrocytes (hematoxylin and eosin stain, x10). (C) High-power view highlighting dense perivascular inflammation with prominent leukocytoclasia and scattered extravasated erythrocytes, consistent with leukocytoclastic vasculitis (hematoxylin and eosin stain, x20). The red arrow indicates leukocytoclasia (neutrophilic debris), and the yellow arrow indicates extravasated erythrocytes.

We present the case of a 26-year-old male with chronic, treatment-refractory immunoglobulin A vasculitis who developed facial and neck swelling, erythema, diffuse rash, and fatigue approximately four weeks after initiation and subsequent dose escalation of dapsone. His vasculitis initially manifested as palpable purpura on his lower extremities and abdominal discomfort. He denied hematochezia or melena. Skin biopsy of the right lower leg revealed leukocytoclastic vasculitis (Figure 1), and direct immunofluorescence demonstrated moderate (2+) perivascular IgA deposition, consistent with immunoglobulin A vasculitis. Histopathology demonstrated a superficial and deep perivascular neutrophilic infiltrate with leukocytoclasia and associated extravasated erythrocytes, consistent with leukocytoclastic vasculitis.

The patient had previously been treated with high-dose systemic corticosteroids, including prednisone 60 mg daily followed by dexamethasone 8 mg daily for 14 days with taper, with inadequate disease control. Due to persistent disease activity, cyclosporine 100 mg twice daily was continued. Despite this regimen, he experienced ongoing and worsening abdominal pain and recurrent palpable purpura, prompting the initiation of dapsone 100 mg daily after confirmation of a normal glucose-six-phosphate dehydrogenase level. This was performed prior to dapsone initiation to reduce the risk of dapsone-associated hemolytic anemia. At the two-week follow-up, the dapsone dose was increased to 100 mg twice daily due to continued active disease.

**Figure 2:**
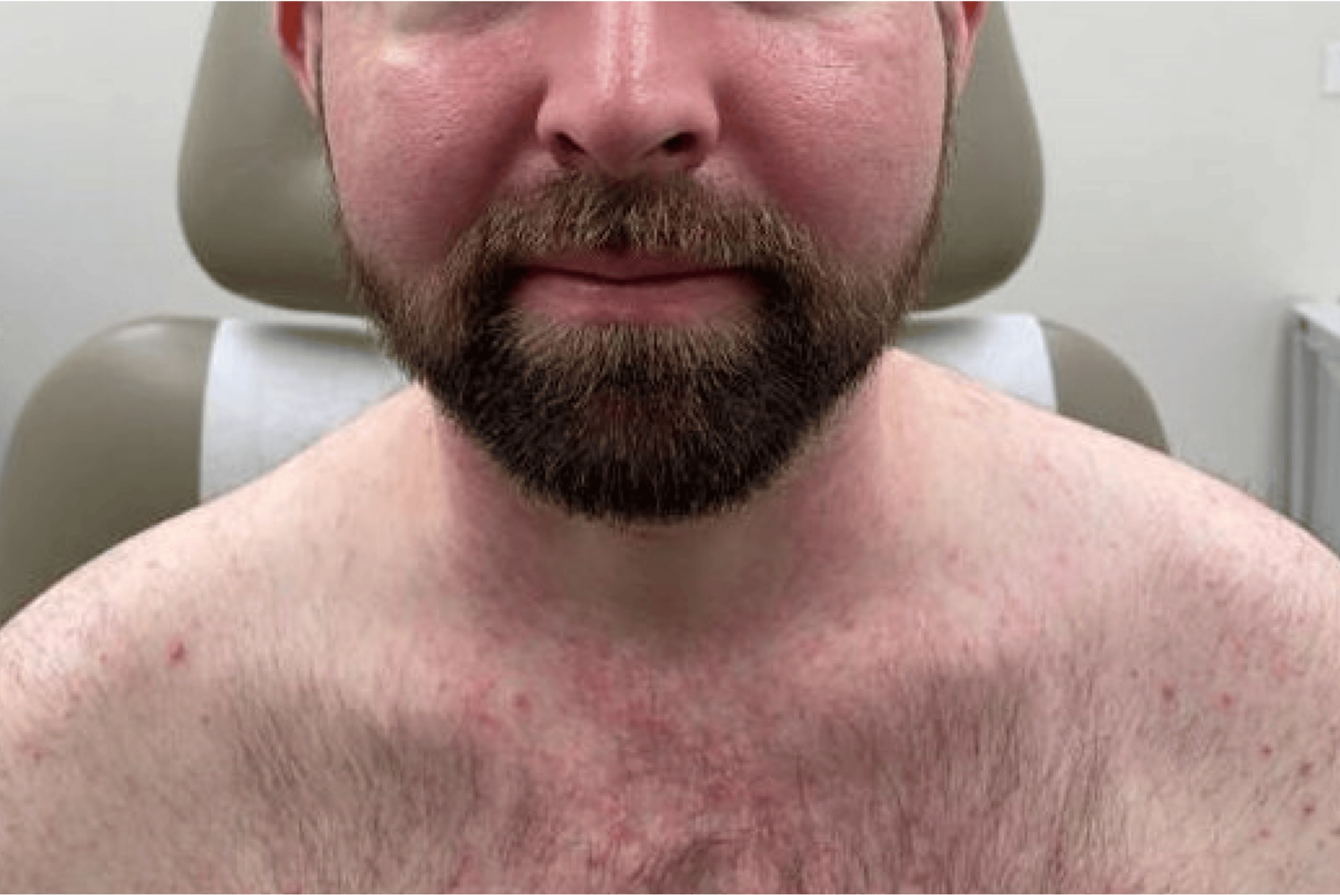
Swelling and erythema of the face at initial presentation.

**Figure 3:**
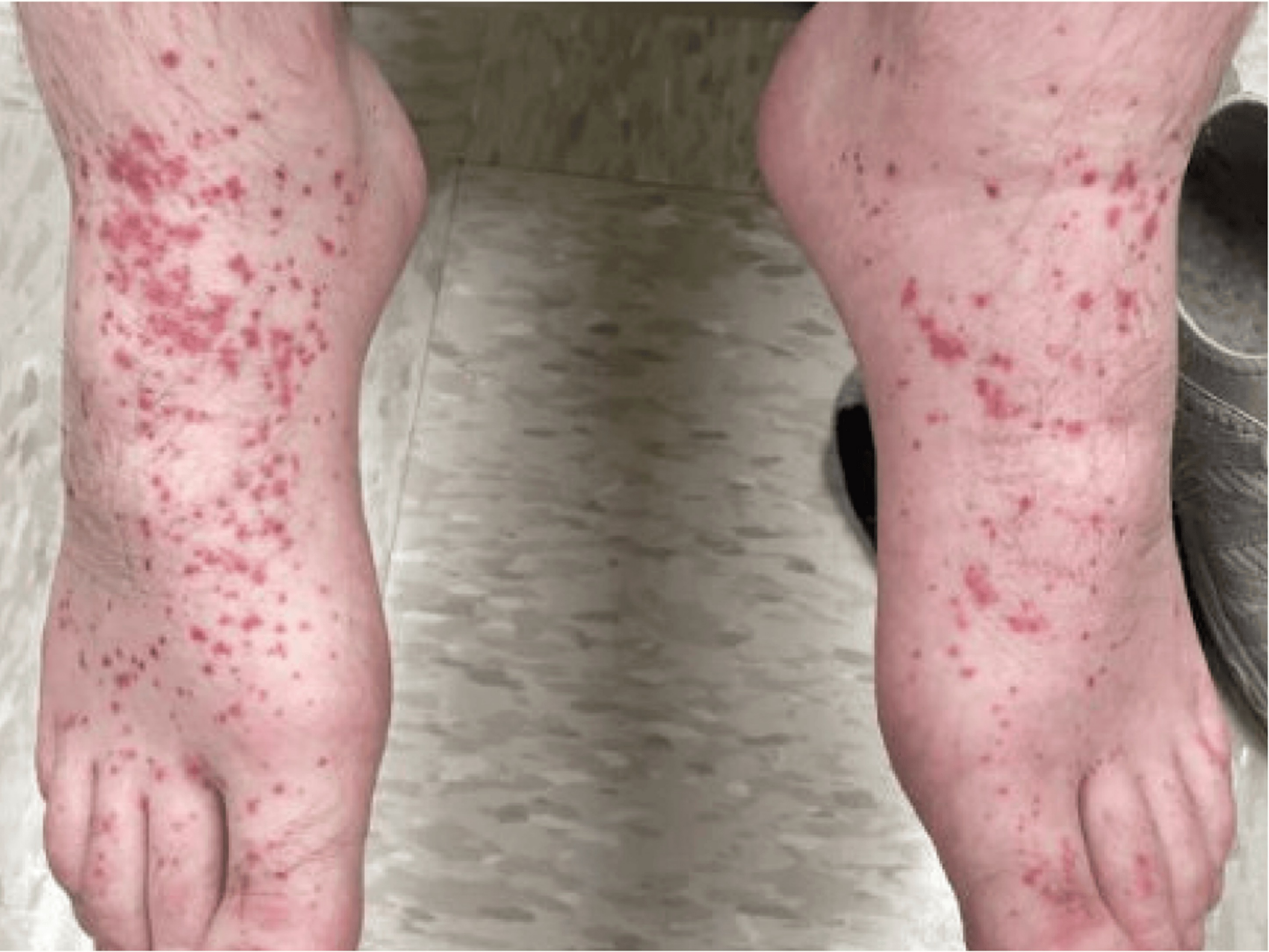
Petechial to small purpuric macules on the bilateral feet at initial presentation.

Approximately one month after dapsone initiation and dose escalation, he developed diffuse facial and neck edema (Figure 2) with erythema and a widespread erythematous morbilliform eruption involving the trunk and extremities, with petechial to small purpuric macules on the lower extremities (Figure 3). He denied mucosal involvement and skin pain. This eruption differed from his baseline vasculitis, which had been limited to palpable purpura of the lower extremities, whereas the new eruption was diffuse, morbilliform, and associated with facial edema and systemic findings.

Laboratory evaluation demonstrated hepatocellular injury, anemia with macrocytosis and reticulocytosis, and no peripheral eosinophilia. He had no evidence of renal involvement, with normal creatinine and no hematuria or proteinuria. He also denied dyspnea or hypoxia, and there was no clinical concern for methemoglobinemia. Leukocyte differential revealed no atypical lymphocytosis. A detailed summary of laboratory investigations is provided in Table 1.

**Table 1:** Laboratory investigations on presentation

Laboratory test	Patient value	Reference range	Units
Alanine aminotransferase (ALT)	193	5–50	U/L
Aspartate aminotransferase (AST)	133	13–40	U/L
Alkaline phosphatase	142	34–122	U/L
Total bilirubin	1.7	0.1–1.1	mg/dL
Hemoglobin	12	12.2–16.4	g/dL
Mean corpuscular volume (MCV)	102	81.7–95.6	fL
Reticulocytes (%)	8.48	0.59–2.24	%
Absolute reticulocyte count	0.2985	0.026–0.117	×10⁶/µL
Eosinophils (%)	0.3	1–4	%
Absolute eosinophil count	<0.03	0.06–0.53	×10³/µL

Given the temporal relationship to dapsone, the presence of facial edema, morbilliform eruption, and hepatic involvement, a severe dapsone-induced systemic drug reaction was suspected. The differential diagnosis included viral exanthem with transaminitis, vasculitis flare with systemic involvement, Stevens-Johnson syndrome, and isolated dapsone-related hematologic toxicity. Dapsone was discontinued immediately, and systemic corticosteroids were restarted with dexamethasone while cyclosporine was continued. The patient underwent close clinical monitoring and exhibited rapid improvement in cutaneous findings and facial edema within one week, with subsequent normalization of liver enzymes and no evidence of relapse on follow-up.

## Discussion

This case illustrates a dapsone-induced systemic hypersensitivity reaction presenting with facial edema, diffuse morbilliform eruption, and hepatocellular injury approximately four weeks after drug initiation and dose escalation. Dapsone-associated severe drug reactions have been well described, including recent case reports and cohort analyses, supporting the plausibility of this presentation and the importance of early drug withdrawal [1].

A central teaching point is the absence of eosinophilia on index laboratory testing. At initial presentation, eosinophils were 0.3% with an absolute eosinophil count < 0.03 × 10³/µL. Although eosinophilia is characteristic of many systemic drug reactions, it is not universally present and does not exclude severe hypersensitivity, particularly early in the disease course or in patients receiving immunosuppressive therapy [3-6]. In this patient, concurrent immunosuppression was present, including ongoing cyclosporine therapy and recent high-dose systemic corticosteroid exposure, both of which plausibly attenuate eosinophil responses and other inflammatory markers. Recent analyses emphasize that severe drug reactions exist along a spectrum and that rigid application of diagnostic criteria may fail to capture atypical or immunosuppression-modified cases [4-6]. While formal causality assessment tools such as the Naranjo scale or WHO-UMC criteria can be applied, the diagnosis in this case was supported by the temporal relationship, characteristic clinical features, and improvement following drug withdrawal.

The differential diagnosis included viral exanthem, vasculitis flare with systemic involvement, Stevens-Johnson syndrome, and isolated dapsone-related hematologic toxicity. The absence of mucosal involvement and skin pain made Stevens-Johnson syndrome less likely. Normal creatinine and lack of renal findings argued against a vasculitis flare involving the kidneys. The temporal relationship to dapsone, characteristic facial edema, morbilliform eruption, and hepatic involvement favored a systemic drug reaction. Concurrent anemia with reticulocytosis suggested overlapping dapsone-related hematologic toxicity, a recognized adverse effect that may coexist with hypersensitivity reactions and further obscure diagnosis [1,7,8].

Management of severe systemic drug reactions centers on immediate withdrawal of the offending agent and supportive care. Systemic corticosteroids remain the most frequently employed therapy in patients with clinically significant organ involvement, particularly hepatitis, although optimal dosing and duration remain debated [4,9-11]. Many patients require prolonged tapers due to relapse risk. In this case, rapid improvement despite a relatively short corticosteroid course likely reflected early recognition, prompt dapsone cessation, limited organ involvement, and background immunosuppression with cyclosporine.

Preventive strategies are particularly relevant for dapsone prescribing. In addition to routine glucose-six-phosphate dehydrogenase testing, genetic susceptibility plays a major role. Multiple studies have demonstrated a strong association between dapsone hypersensitivity reactions and the HLA-B*13:01 allele. Some genome-wide association studies have shown that the presence of this allele is associated with a 20-fold increased risk of dapsone hypersensitivity reaction and sensitivity of 85.5% and specificity of 85.7% for predicting the drug reaction [12-14]. Mechanistically, dapsone is metabolized to reactive hydroxylamine derivatives that bind host proteins and form immunogenic complexes, triggering T-cell-mediated immune responses in genetically susceptible individuals [12,15]. Selective human leukocyte antigen testing may therefore be considered in higher-risk populations or when alternative therapies are available.

## Conclusions

Dapsone can precipitate a clinically significant systemic drug reaction characterized by facial edema, morbilliform eruption, and hepatic injury, even in the absence of eosinophilia. This case highlights the importance of integrating medication timelines, characteristic clinical features, and evidence of organ involvement rather than relying on a single laboratory criterion, particularly in patients receiving concurrent immunosuppression or with overlapping underlying dermatologic conditions.

Prompt discontinuation of the offending agent, careful laboratory evaluation including absolute eosinophil count and renal indices, and close clinical follow-up are essential to achieving favorable outcomes. Clinicians who prescribe dapsone should ensure glucose-six-phosphate dehydrogenase testing and appropriate baseline and periodic monitoring, including complete blood count and liver function tests, and remain aware of emerging data on genetic susceptibility when selecting therapy in higher-risk patients.
